# Longitudinal Study of *TCF4* CTG Trinucleotide Repeat Length and Disease Severity in Fuchs’ Endothelial Corneal Dystrophy

**DOI:** 10.3390/medsci14010031

**Published:** 2026-01-07

**Authors:** Jasmin X. J. Teo, Dawn J. H. Neo, Jessica Q. H. Choo, Xin Gong, Zheng Li, Hla Myint Htoon, Min Jie Chua, Yu Qiang Soh, V. Vinod Mootha, Chiea Chuen Khor, Jodhbir S. Mehta

**Affiliations:** 1 Singapore Eye Research Institute, Singapore 169856, Singapore; jasmin.teo@mohh.com.sg (J.X.J.T.); dawn.neo.j.h@seri.com.sg (D.J.H.N.); jessica.choo.q.h@singhealth.com.sg (J.Q.H.C.); hla.myint.htoon@seri.com.sg (H.M.H.); chuaminjie071@gmail.com (M.J.C.); yu.qiang.soh@nhghealth.com.sg (Y.Q.S.); khorcc@gis.a-star.edu.sg (C.C.K.); 2 Singapore National Eye Centre, Singapore 168751, Singapore; 3 Yong Loo Lin School of Medicine, National University of Singapore, Singapore 117597, Singapore; 4 Department of Ophthalmology, University of Texas Southwestern Medical Center, Dallas, TX 75390, USA; xin.gong@utsouthwestern.edu (X.G.); vinod.mootha@utsouthwestern.edu (V.V.M.); 5 Genome Institute of Singapore, Agency for Science, Technology and Research, Singapore 138672, Singapore; liz11@gis.a-star.edu.sg; 6 Ophthalmology and Visual Sciences Academic Clinical Programme, Duke-NUS Medical School, Singapore 169857, Singapore; 7 Eugene McDermott Center for Human Growth and Development, University of Texas Southwestern Medical Center, Dallas, TX 75390, USA

**Keywords:** CTG trinucleotide repeat expansion, Fuchs’ endothelial corneal dystrophy, TCF4 gene

## Abstract

**Objective:** This was a longitudinal study of *TCF4* CTG18.1 trinucleotide repeat lengths in 17 patients (27 eyes) diagnosed with Fuchs’ endothelial corneal dystrophy (FECD), and it aimed to correlate the repeat expansion status with disease severity and progression. **Design:** This was a prospective cohort study looking at FECD clinical progression and *TCF4* CTG18.1 repeat length expansion status over time. **Methods:** A total of 27 eyes from 17 patients diagnosed with FECD were recruited. Only eyes with FECD disease severity of at least Grade 4 on the modified Krachmer clinical grading scale were included; eyes that had previously undergone any form of ocular surgery prior to the first genotyping or during the duration of follow-up were excluded. CTG trinucleotide repeat genotyping was performed on peripheral blood leukocytes at two time points over an average follow-up of 10 years. Over the follow-up period, the FECD progression of each subject was examined using pachymetry, Scheimpflug imaging (Pentacam), and endothelial cell density (ECD) readings, during the baseline visit, yearly thereafter, at the time of repeat CTG18.1 genotyping, and at their latest visit. **Main Outcome Measures:** The clinical progression of FECD patients was assessed using central corneal thickness (CCT), ECD, and any keratoplasty performed. CTG repeat length was assessed twice over the entire follow-up period. **Results:** The non-expanded alleles were shown to be stable over the period of follow-up and did not develop any expanded repeats. Repeat expansion did not influence the risk of attaining Threshold Disease, although more patients in the L ≥ 40 group (CTG18.1 repeat sequence of more than or equal to 40 repeats) underwent keratoplasty. **Conclusions:** Through this study, we found that the CTG18.1 allele lengths of <40 repeats in peripheral blood leukocytes showed minimal change over a 10-year period, and none became an expanded repeat. Hence, a single CTG expansion assessment, performed at any point in a patient’s lifetime, is likely a good representation of genetic risk. Clinicians may use this information to better advise patients on the risk of clinical progression and the best therapeutic strategy.

## 1. Introduction

Fuchs’ endothelial corneal dystrophy (FECD) is one of the leading indications for cornea transplantation worldwide [1]. It causes progressive endothelial cell loss and posterior focal excrescences on the Descemet’s membrane. These clinical signs, including corneal oedema (due to endothelial cell dysfunction), may present in the fourth to fifth decades of life, with early symptoms, including haloes, glare, and blurring of vision, manifesting afterwards. In the later stage of the disease, due to further endothelial dysfunction, the corneal oedema worsens, leading to further loss of vision and ultimately blindness without interventions. FECD is estimated to have a prevalence rate of 7.33% worldwide, affecting nearly 300 million people above 30 years old. This number is anticipated to increase by 41.7% to 415 million by the year 2050 [2].

FECD is a complex genetic disease with environmental factors [3]. One of the first mutations identified was in the *COL8A2* gene, which codes for collagen type VIII, a major component of the Descemet’s membrane [4]. Since then, various variants in different genes have been identified to be linked to FECD, such as *AGBL1* [5], *LOXHD1* [6], and *SLC4A11* [7]. However, the most prevalent genetic association identified thus far is an expansion in the intronic CTG trinucleotide repeat polymorphism (CTG18.1) within the *TCF4* gene. The association was first reported in a study in 2012 by Wieben et al., where 79% of patients with FECD had trinucleotide repeat lengths of more than 50, compared to 3% in normal individuals [8]. Transethnic replication of the association of the *TCF4* CTG18.1 repeat expansion with FECD in a Singapore Chinese population in 2014 by Xing et al. established the expansion as a causal variant [9]. An expanded CTG18.1 allele conferred a 66-fold increased risk of developing FECD in this Singapore Chinese cohort [9]. Another study in 2018 conducted on participants of European ancestry showed that the trinucleotide repeat expansion brought about a 76-fold increased risk of developing FECD [10]. Cross-ancestry analyses have revealed that the prevalence of CTG18.1 repeat expansion in FECD patients also differs according to ethnicity. For example, the prevalence is higher (79%) in Germans [11], but less in the Asian population—44% in the Singapore Chinese cohort [9], 34% in Indians [12], and only 26% in Japanese [13].

Trinucleotide repeat expansion disorders are a group of diseases where abnormal expansion of gene sequences leads to clinical disease, commonly neurodegenerative [14]. The mechanisms behind FECD disease pathogenesis related to the *TCF4* CTG18.1 expansion have been postulated to be similar to other repeat expansion disorders [15]. For instance, this includes somatic instability of DNA, RNA-mediated toxicity, and dysregulation of gene expression [16]. The presence of a trinucleotide repeat expansion in the *TCF4* gene has a 79% sensitivity and a 96% specificity for FECD, allowing one to use this factor to predict the risk of disease [8].

The presence of pathologically long trinucleotide repeat expansions may also be associated with disease severity. In FECD patients with *TCF4* CTG18.1 repeat expansions, the modified Krachmer grade has been shown to be more severe than that of those without [17]. Previously, we reported that those patients with repeat expansions also showed faster clinical progression at least in the first 5 years compared to those without [18]. This is likely due to the fact that expanded alleles lead to higher levels of instability and thus worse clinical outcomes [19,20]. It has also been shown in a study by Eghari et al. that CTG18.1 repeat expansion led to a 1.64 times increased likelihood of cornea transplantation in FECD [21].

Despite the many studies on the association of the CTG18.1 repeat expansion with FECD, there has been little research evaluating the status of CTG repeat expansion longitudinally over time. In this study, we evaluated CTG repeat expansion in the peripheral blood leukocytes of 17 FECD patients by looking at changes in repeat length over time. We also compared data on disease severity and progression in relation to the CTG repeat length. This would help us to understand how one’s genetic risk changes over a lifetime and how this may influence disease progression.

## 2. Methods

This was a prospective cohort study of 27 eyes from 17 patients at the Singapore National Eye Centre diagnosed with FECD. Diagnosis was based on classical clinical outcomes, such as corneal guttae (excrescences seen on the Descemet’s membrane) and corneal oedema, visible through slit lamp biomicroscopy, and anatomical assessment of the cornea endothelium by looking at the endothelial cell density, morphology (pleomorphism), and size (polymegathism) of corneal endothelial cells through specular microscopy [22,23]. The inclusion criteria were that of FECD with disease severity of at least Grade 4 on the modified Krachmer clinical grading scale, which is given by the following—Grade 0: no guttae; Grade 1: 1–12 central/paracentral non-confluent guttae; Grade 2: >12 central/paracentral non-confluent guttae; Grade 3: 1–2 mm of confluent central/paracentral guttae; Grade 4: >2 to 5 mm of confluent central/paracentral guttae; Grade 5: >5 mm confluent central/paracentral guttae; and Grade 6: >5 mm of confluent central/paracentral guttae with stromal and/or epithelial oedema [24,25]. All patients were recruited after providing informed consent, with an explanation of the nature and possible consequences of this study. Seventeen patients were recruited from January 2005 to January 2016. Data from each eye of the patients was considered for this study, except for eyes that underwent any form of ocular surgery prior to the first genotyping or during the course of follow-up. Exclusion of eyes that underwent ocular surgery, such as cataract extraction, was deemed necessary, as it variably accelerates corneal endothelial cell loss and possibly the progression of FECD [26]. A total of 27 eyes were eligible for analysis from the 17 patients. All patients had genotyping of the *TCF4* CTG18.1 trinucleotide repeat polymorphism performed twice over two separate time points. Measurements of central corneal thickness (CCT) and endothelial cell density (ECD) were obtained at baseline, yearly, at the time of repeat CTG18.1 genotyping, and at the patient’s last visit. This study was performed in accordance with the tenets of the Declaration of Helsinki, with ethical approval granted by the Singapore Health Services (SingHealth) Institutional Review Board (IRB).

### 2.1. Genotyping

Genotyping of the *TCF4* CTG18.1 trinucleotide repeat polymorphism was performed in a manner similar to that described previously [27,28]. A total of 10 mls of venous blood was obtained from each patient upon enrolment into this study, and a FlexiGene DNA kit (QIAGEN, Germantown, MD, USA) was used to extract genomic DNA from peripheral leukocytes. Both short tandem repeat (STR) analysis and triplet primed polymerase chain reaction assays (TP-PCR) were performed. While STR analysis was performed for all subjects, TP-PCR was performed to confirm the presence of an expanded allele when STR analysis detected only one allele or failed to detect any alleles [27]. In accordance with a previous study of FECD patients in a local population, an expanded allele was defined as one harbouring a trinucleotide repeat sequence with ≥40 CTG repeats [9]. Patients with at least one allele in the CTG18.1 locus whose length was equal to or greater than 40 repeats were labelled as ‘L ≥ 40’, while those with both alleles having fewer than 40 repeats were labelled as ‘L < 40’.

STR analysis was limited by its ability to provide exact quantification of CTG repeat lengths only for alleles with fewer than ~100 CTG repeats; Southern blot analysis would have been necessary to ascertain the exact number of CTG repeats for alleles containing more than 100 repeats. However, in accordance with our study design of grouping patients into the dichotomised groups of L ≥ 40 vs. L < 40, such a high level of precision in determining CTG repeat frequency was not required; thus, Southern blot analysis was not performed.

Whole-genome sequencing was also performed at the repeat blood draw to confirm the DNA sequences against the STR analysis results. Sequencing libraries were prepared using genomic DNA obtained from all consenting participants. Illumina TruSeq PCR-free kits (Illumina, Inc., San Diego, CA, USA) were used for maximum fidelity. Each sequencing library was subjected to quality checks before being sequenced using 2 × 151 base pair chemistry on an Illumina Novaseq 6000 (Illumina, Inc., San Diego, CA, USA). Genomic DNA was subjected to 30X whole-genome sequencing.

All raw DNA sequence reads were aligned to the hg38 genome build. Aligned reads duplicating the start position of another read were flagged as ‘duplicates’ and excluded from further analysis. To avoid erroneous findings, all data was processed uniformly from the beginning by aggregating BAM files from unaligned sequence reads using PICARD, Burrow-Wheeler Aligner (BWA), and Genome analysis tool kit (GATK) software packages, following best practice guidelines [29]. Genetic variants were identified and genotyped using recalibrated BAM files and the Haplotype caller from the GATK. The *TCF4* CTG18.1 tandem repeat was genotyped using short-read whole-genome sequencing. Expanded alleles (repeat number more than or equal to 40) were denoted as X, and non-expanded, normal alleles (repeat number less than 40) were denoted as S. The genotypic value was coded in an additive manner, with SS, SX, and XX genotypes denoting the CTG18.1 polymorphisms.

### 2.2. Central Corneal Thickness and Endothelial Cell Density

Central corneal thickness (CCT) was measured with an ultrasound pachymeter (Sonogage Inc., Cleveland, OH, USA) and Scheimpflug Pentacam (Oculus Inc., Arlington, WA, USA), while endothelial cell density (ECD) at the centre of the cornea was measured with a non-contact specular microscope (Konan Medical Corp., Hyogo, Japan), using the Center Method. When only a small number of endothelial cells were visible, the Flex-Center method was used. Qualified ophthalmic medical technicians were employed by the Singapore National Eye Centre to acquire these measurements. The value of each CCT and ECD measurement was taken as the mean of three consecutive readings. All measurements were acquired between 10 am and 3 pm to reduce confounding secondary to diurnal variations in CCT. Pachymetry, Pentacam, and ECD readings were obtained for all patients during the baseline visit, yearly thereafter, at the time of repeat CTG18.1 genotyping, and at the final visit.

### 2.3. Clinical Progression

All patients were reviewed at least once per year. The examined eye was defined to have experienced significant clinical progression from baseline, thus achieving the status of ‘Threshold Disease’, if at the time of assessment, any one of the following three criteria was fulfilled: (a) undergone keratoplasty, (b) CCT greater than 700 μm, or (c) central ECD less than 700 cells/mm^2^.

### 2.4. Statistical Analysis

All statistical analyses were performed using the GraphPad QuickCalcs Website: http://www.graphpad.com/quickcalcs/ (accessed on 10 May 2024). A post hoc power analysis was performed using PASS 2022 Power Analysis and Sample Size Software (2022) (NCSS, LLC., Kaysville, UT, USA, ncss.com/software/pass) [30,31]. In terms of descriptive statistics, for continuous variables, the mean and standard deviation were calculated; for categorical variables, frequency distribution and categorical percentages were calculated. The unpaired *t*-test and the Fisher exact test were used to compare means and categorical distributions, respectively. A *p*-value of less than or equal to 0.05 was considered statistically significant.

## 3. Results

### 3.1. Demographics

A total of 27 eyes from 17 patients were analysed for this study. In total, 3 out of 16 eyes were excluded from the L < 40 group, and 4 out of 18 eyes were excluded from the L ≥ 40 group; therefore, approximately the same ratio of eyes was excluded from both groups. The patients’ ages ranged from 46.6 to 77.0 years old at the time of first blood draw from January 2005 to January 2016, with an average age of 57.2 ± 7.18 years old. Overall, 58.8% (*n* = 10) were females and 70.6% (*n* = 12) were of Chinese ancestry, with the rest being of Malay (*n* = 3), Indian (*n* = 1), and Sikh (*n* = 1) ancestry. For 10 patients, data from both eyes was available. The patients were followed-up over an average duration of 10.7 years (range: 4.7 to 15.8 years). The demographics of the patients are summarised in Table 1.

### 3.2. Longitudinal Change in CTG18.1 Expansion Length

**Figure 1:**
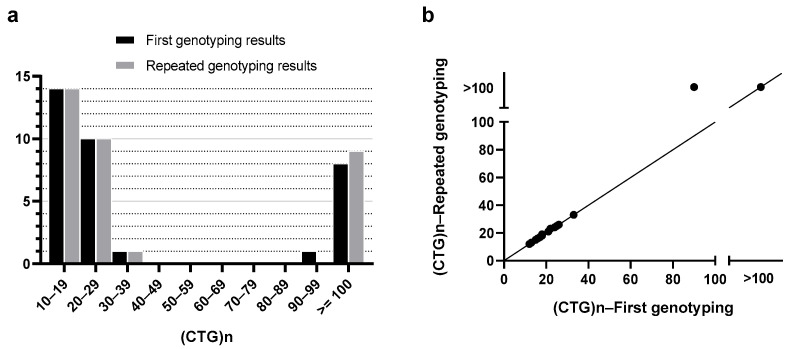
Graphs showing the distribution of (CTG)n in peripheral blood leukocytes of FECD patients at first and repeated genotyping. (**a**) Histogram comparing patients’ CTG18.1 allele repeat lengths at first and repeated genotyping. (**b**) Scatter plot showing discrepancy for 1 patient with an increase in repeat lengths. The prevalence of L < 40 and L ≥ 40 was the same at the first and second blood genotyping.

The first blood draw was performed during the period from January 2005 to January 2016. Overall, 51.9% of the patients’ eyes (*n* = 14) had CTG18.1 repeat lengths of more than or equal to 40 repeats (L ≥ 40) at the first blood test typing, while 48.1% (*n* = 13) had CTG18.1 repeat lengths of less than 40 (L < 40). The repeat blood test was performed from the period of September 2020 to January 2021, where the prevalence of L ≥ 40 and L < 40 was found to remain the same. The average number of years between the first and second blood tests was 10.62 ± 3.71 years for the L < 40 group and 9.78 ± 4.31 years for the L ≥ 40 group; thus, the duration of follow-up between the two groups was comparable (*p* = 0.594). The comparison between the number of CTG repeats at first and repeat peripheral blood leukocyte genotyping, and the respective scatter plot representing this data, is shown in Figure 1. The representative STR/TP-PCR tracings of the genotyped samples can be seen in Supplementary Figure S1.

With these results, we evaluated whether the *TCF4* CTG18.1 tandem repeat could be genotyped using short-read whole-genome sequencing. The 17 patients from our study who were assayed for CTG18.1 using the previously reported gold-standard method (combination of short tandem repeat (STR) analysis and triplet repeat primed PCR assay) were subjected to 30× whole-genome sequencing. The concordance rate was 17 out of 17 samples (100.0%), as shown in Table 2, suggesting that *TCF4* tandem repeat status could be reliably identified and confirmed using routine whole-genome sequencing [32]. The details of each individual patient are summarised in Table 3.

### 3.3. Clinical Progression

**Figure 2:**
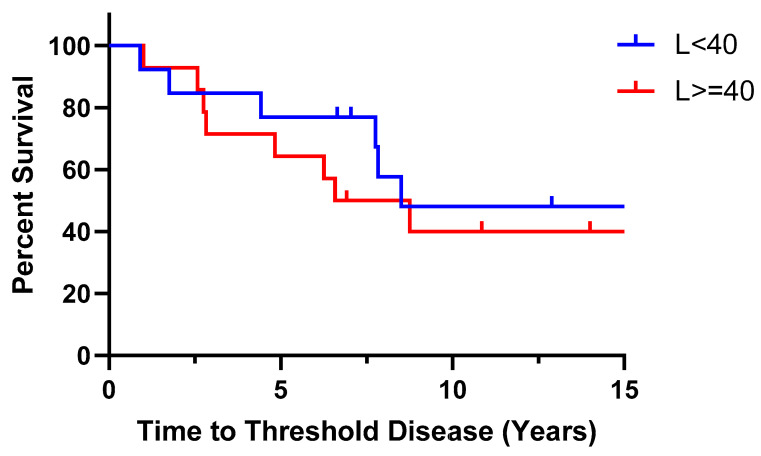
Kaplan–Meier time-to-event curve showing the time to Threshold Disease for patients with L < 40 and L ≥ 40. Threshold Disease was determined when a patient (1) underwent keratoplasty, (2) had CCT greater than 700 µm, or (3) had ECD that dropped below 700 cells/mm^2^. No patients achieved Threshold Disease through CCT > 700 µm. There was no significant difference between survival curves using the Log-rank (Mantel–Cox) test (*p* = 0.7130).

**Figure 3:**
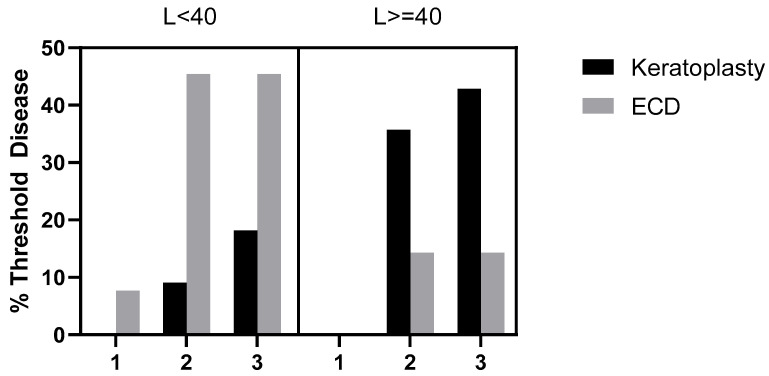
Bar chart showing percentage of patients in both the L < 40 and L ≥ 40 groups who attained Threshold Disease status at 3 time points—(1) at the first blood test, (2) at the second blood test, and (3) 2 years after the second blood test. Threshold Disease status is classified by the following criteria: having undergone keratoplasty, CCT > 700 µm, or having an endothelial cell density (ECD) of less than 700 cells/mm^2^. None of the patients reached Threshold Disease with CCT > 700 µm.

Clinical information for 27 eyes and 25 eyes was available at the points of the first blood test and the second blood test, respectively, as one patient in the L < 40 group was lost to follow-up in 2016. At the point of the first blood test, only one of the eyes (7.69%) in the L < 40 group had Threshold Disease as ECD counts reached less than 700 cells/mm^2^, while none of the patients in the L ≥ 40 group were at Threshold Disease (*p* = 0.482). At the point of the second blood test, 54.5% (*n* = 6) of the L < 40 group had reached Threshold Disease, which was comparable to 50.0% (*n* = 7) in the L ≥ 40 group (*p* = 1.0). Of the six eyes in the L < 40 group that reached Threshold Disease, five of them (83.3%) fulfilled ECD count criteria of less than 700 cells/mm^2^, while one (16.7%) underwent keratoplasty. In the L ≥ 40 group, a larger proportion of 71.4% (*n* = 5) underwent keratoplasty, while two (28.6%) fulfilled ECD count criteria (*p* = 0.103). After the second blood test, 25 eyes were continually followed up. Two years after the second blood test, 63.6% (*n* = 7) reached Threshold Disease in the L < 40 group vs. 57.1% (*n* = 8) in the L ≥ 40 group (*p* = 1.0). The additional eye that reached Threshold Disease in each group underwent keratoplasty. During the period of follow-up, no patient reached Threshold Disease by means of fulfilling the criterion of CCT > 700 μm. The patients in the L < 40 group took an average of 7.63 years to reach Threshold Disease (range 1.75 to 15.50 years), longer compared to the patients in the L ≥ 40 group, who took an average of 4.45 years (range 1.00 to 8.75 years) (*p* = 0.127). Comparing the proportion of patients who reached Threshold Disease within 5 years in the two groups, it was higher in the L ≥ 40 group, at 35.7% (*n* = 5), compared to 16.7% (*n* = 2) in the L < 40 group (*p* = 0.391). This result is similar to our centre’s previous study, where the disparity in patients reaching Threshold Disease between the two groups was most significant at the 5-year mark [18]. This can also be seen in Figure 2, where a Kaplan–Meier time-to-event curve shows a slightly steeper curve in the first 5 years. The median time-to-event taken was 8.5 years for L < 40 and 7.665 years for L ≥ 40. Using the Log-rank (Mantel–Cox) test, there was no significant difference between survival curves in the Kaplan–Meier time-to-event curve (*p* = 0.7130). The percentage of patients who had significant clinical progression and reached Threshold Disease, and the criteria that they fulfilled, can be seen in Figure 3. At all three time points, the difference in Threshold Disease proportions in the two groups did not reach statistical significance.

**Figure 4:**
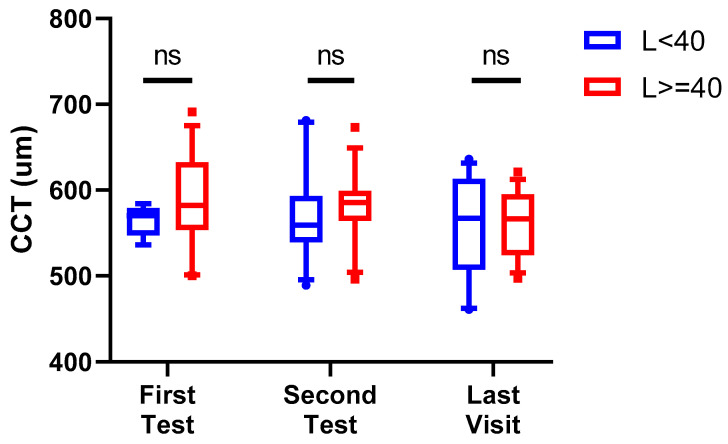
Distribution of central cornea thickness (CCT) (µm) of patients at the first test, second test, and last visit date. No patients attained Threshold Disease status with CCT greater than 700 µm. Mean CCT for L < 40 at the first blood test, second blood test, and last visit dates were 563.778 ± 17.740, 574.545 ± 58.187, and 561.000 ± 60.329 µm, respectively. Mean CCT for L ≥ 40 at the first blood test, second blood test, and last visit dates were 588.143 ± 58.045, 580.500 ± 43.687, and 561.500 ± 38.422 µm, respectively. There was no significant difference between the mean CCT of L < 40 and L ≥ 40 patients (*p* = 0.4642, 0.9983, and 0.9999 for each time point, respectively).

The distribution of CCT values in the L < 40 and L ≥ 40 groups at the first blood test, at the second blood test, and at the latest visit (ranging from July 2021 to January 2023) is plotted in Figure 4. The mean CCT values for L < 40 at the first blood test, the second blood test, and the last visit dates were 563.778 ± 17.740, 574.545 ± 58.187, and 561.000 ± 60.329 µm, respectively. The mean CCT values for L ≥ 40 at the first blood test, the second blood test, and the last visit dates were 588.143 ± 58.045, 580.500 ± 43.687, and 561.500 ± 38.422 µm, respectively. There was no significant difference between the mean CCT of L < 40 and L ≥ 40 patients at all visit dates (*p* = 0.4642, 0.9983, 0.9999 for each time point, respectively).

## 4. Discussion

There have been multiple papers describing the association of the *TCF4* CTG18.1 trinucleotide repeat expansion with FECD; however, none have analysed the longitudinal change in CTG18.1 repeat length and disease severity concurrently in FECD patients over time through a prospective cohort study. Our results showed that the proportion of patients with CTG18.1 repeats L < 40 and L ≥ 40 in peripheral blood leukocytes remained the same at the two time points of analysis. Thus, one’s CTG18.1 allele lengths of <40 CTG repeats in peripheral leukocytes and genetic risk for FECD are likely to show minimal change in one’s lifetime, i.e., there is stability within the non-expanded allele.

Although patients with L < 40 may have different genetic risk factors and may be a heterogeneous group in relation to severity and progression, we previously reported that the presence of the CTG repeat expansion in our Singapore FECD cohort was a good predictor for future clinical progression. We found patients with L < 40 tend to have less severe disease with slower progression compared to L ≥ 40 patients [9,18,20]. Thus, determining whether TCF4 CTG trinucleotide repeat length is <40 or ≥40 still gives a good prediction of the severity and progression of the disease. Through this study, we found that a single CTG repeats assessment, performed at any point in a patient’s lifetime, is likely to be a good representation of genetic risk, and clinicians may use this information to better advise patients on the risk of clinical progression in our patient population and advise on appropriate treatment options.

There are numerous other diseases that are also caused by trinucleotide repeat expansion, commonly neurodegenerative [14]. It is noted that with most of these diseases, a larger repeat expansion is usually correlated with earlier onset and greater severity of disease [33,34]. It is also known that such diseases tend to show anticipation, by which there is worsening severity of disease with each successive generation [35]. However, with regard to longitudinal change in repeat sequences, there is less data available. For Huntington’s disease, which is related to CAG trinucleotide repeats, studies in mice have shown that CAG repeats were stable over time in more stable tissues, such as spleen and tail, but in unstable tissues, such as liver and striatum, unstable repeats showed continuous expansion over time, while no significant shift was observed in the constitutive repeat [36]. Other studies in humans also showed that mutant CAG repeats lengthen progressively with time in the brain, while the normal CAG repeat displays less somatic instability [37]. A study on CTG repeats in myotonic dystrophy patients also showed that in most of the patients, the length of the repeats in peripheral leukocytes increased with age, and this change was length-dependent, as it was more evident in CTG repeats > 150 compared to those <100 [38]. Another study showed that CTG repeat expansion was also tissue-specific, as it was more frequent in skeletal tissue compared to leukocytes in blood [39]. Similarly, a study of GAA repeats in Friedreich’s ataxia showed progressive expansion with age, and it was also tissue-dependent [40]. These studies suggest that trinucleotide repeat expansions display somatic instability over time in a length-dependent and tissue-dependent manner.

All the subjects in this FECD cohort with the *TCF4* CTG18.1 repeat expansion in their peripheral leukocytes had an expanded allele with a repeat length beyond the upper limit of accurate sizing using STR analysis (~100 CTG repeats). Future studies of the longitudinal changes in large expanded CTG18.1 alleles in peripheral leukocytes should utilise Southern blot analysis or long-read DNA sequencing to assess any changes in the expanded allele. However, our data has shown that in heterozygous patients and patients with no expansion, the short repeat allele is stable over time and does not become expanded (≥40 repeats) with age. This is important in our understanding of the genetic risks associated with CTG repeat expansion—there is little possibility of developing a repeat expansion from a non-expanded allele, which indicates that an initial genetic risk assessment is a good indicator of future disease progression and can be used in determining suitable treatment options catered to the individual.

In addition, our study only analysed CTG repeat lengths from DNA extracted from peripheral leukocytes. Recent publications have shown that the accumulation of toxic CUG repeat RNA transcripts as nuclear foci in FECD subjects with the expansion is tissue-dependent, with the foci being much more abundant with greater repeat lengths in the cornea endothelium [41] compared to other tissues, such as trabecular meshwork and stroma [42]. While it would be interesting to compare changes in CTG repeat lengths of DNA in other tissues for future studies, despite the information that the afflicted tissue (in this case, the cornea endothelium) can provide, the use of corneal tissue as a biomarker is not feasible due to its decreased accessibility as compared to peripheral blood. Due to the nature of FECD, obtaining diseased corneal endothelium is only possible through keratoplasty, which is only carried out at later stages of disease and, therefore, impractical as a measure for predicting severity and progression. It is also not possible to retrieve corneal tissue repeatedly over multiple time points, and, thus, it is impossible to study longitudinally. Furthermore, sizing repeat sequences in endothelial cells from corneal tissue may be difficult due to low ECD and poor viability of cells, leading to poor sample quality. Therefore, in order to provide the clinician with enough data to make an informed decision on the best therapeutic strategy, it would be more feasible to use peripheral blood as a biomarker for FECD CTG expansion, which has been shown to be able to give a good prediction of disease severity and progression [18].

In this study, Threshold Disease was defined as meeting any one of the following three criteria: keratoplasty, CCT greater than 700 μm, or central ECD less than 700 cells/mm^2^ [18]. Visual acuity was not used as an assessment criterion, as it could be confounded by other ocular diseases, most commonly, for example, cataracts. Central corneal thickness (CCT) was found to be positively correlated with the severity of FECD, and the cut-off of 700 μm was chosen as it usually indicates severe disease at this level [25,43]. However, this is confounded by the baseline CCT value, as there is inter-individual variability, and some patients may never reach 700 μm, even in advanced disease if baseline CCT is thin. CCT measurements were acquired between 10 am and 3 pm for all our patients to reduce confounding secondary to diurnal variation. ECD is also another important factor correlated to the disease severity of FECD, where ECD counts become lower as the disease becomes more advanced [44]. Central ECD counts < 700 cells/mm^2^ may not always be a reliable biometric, as, in some cases, central ECD may be low with confluent guttae, but only minimal or subclinical oedema is present if the guttae are small and pump function still persists. Subclinical oedema is best examined by looking at posterior float changes on a Scheimpflug device. Unfortunately, due to the duration of this study, this test was not performed on the initial patients since the device was not available, and thus, it was omitted from our study this time. However, this is now included as standard of care, and it will be considered in future studies to further enhance consistency of the subclinical oedema assessment [43,45]. Although ECD < 700 cells/mm^2^ may not always lead to significant corneal oedema requiring a graft, as the decision to perform a graft is also related to other variables, such as photosensitivity, it is still a threshold that is observed to be associated with more severe disease and cornea decompensation [46,47]. Thus, we included ECD counts as a third criterion for Threshold Disease in addition to CCT and keratoplasty. In this study, we looked only at central cornea ECD, which may have its limitations, especially in severe disease when there is significant loss of cells in the centre. Our centre recently acquired the machine to measure peripheral ECD from widefield peripheral specular microscopy images [48,49]. As there is regional variability between the different zones of the cornea in patients with FECD, peripheral ECD is another variable worth measuring and analysing in the future [50]. However, as this is a new instrument, and we similarly did not have the measurements at baseline for the patients, it was omitted from our study this time. Last but not least, we included patients who required keratoplasty due to advanced disease. However, even in patients who are clinically indicated for keratoplasty, some may choose not to undergo it due to other factors, such as costs and patients’ beliefs, which would influence their decision. In view of the confounding factors for each, we decided to take all three criteria into consideration to define a patient with Threshold Disease [18].

The eyes were also analysed separately due to asymmetry in disease progression for each eye; therefore, meeting the threshold in one eye may not equate to a similar disease severity in the contralateral eye. We also chose to exclude eyes that had undergone prior ocular surgery, as the inclusion of these eyes would provide a confounding effect—ocular surgery could potentially speed up FECD progression. While this exclusion in itself may provide a confounding effect by biasing the cohort towards less severe cases, the number of eyes excluded from each group for this study (3 out of 16 in the L < 40 group, and 4 out of 18 in the L ≥ 40 group) is of a similar ratio. We included two eyes from 10 subjects, and only one eye from the remaining 7 subjects. Although there may be some confounding effects due to inter-eye correlations, all eyes that were eligible were included in this study to increase the sample size.

Our analysis showed that there was a high degree of concordance between the results of short-read whole-genome sequencing (WGS) compared to STR with TP-PCR. STR analysis with TP-PCR has long been the gold standard for analysis of DNA repeat expansions [51,52]. However, it does have its limitations, for example, not being able to provide accurate sizing of large repeat expansions and also being a time-consuming procedure to perform. It is also only able to analyse one or a few target loci at a time. For this study, we determined that the accurate detection of upper limits of CTG repeats > 100 was not necessary due to the dichotomisation of our patients into the L < 40 and L ≥ 40 groups, since the severity of FECD is determined by the presence of repeat expansion rather than the exact length of expansion [18]. For future studies, the use of more accurate sizing methods can be employed, such as short-read WGS, which has now become widely available in most labs and is less tedious to perform compared to STR [53,54]. Although there are concerns regarding the detection of repeat expansion disorders accurately with short-read WGS methods, our data shows that it is a reliable method to substitute STR + TP PCR. Thus, the use of short-read WGS for the analysis of repeat expansions could be employed instead for greater efficiency, while STR + TP-PCR can be used to validate samples indicated as expansion-positive by WGS.

Based on methods employed in this study, we determined that patients who do not have repeat expansion will unlikely develop expansions over time, which has not been shown before for FECD patients specifically. However, it is noted that due to technical limitations in methodology, the use of STR + TP-PCR in sizing the CTG18.1 repeat lengths cannot ascertain repeats above 100 in number. Therefore, the longitudinal stability of large expanded alleles is inconclusive at this stage. Despite this, this study showed that patients with non-expanded repeats will not cross the threshold to become expanded, and that a single assessment of the genetic risk of the patient will suffice as a predictor of future progression of disease.

The percentage of patients who reached Threshold Disease at the second blood test and 2 years after the second blood test was slightly higher in the L < 40 group compared to the L ≥ 40 group. However, the results are comparable with only a small difference. In this study, our results did not show that the presence of expanded repeats within peripheral blood leukocytes influenced the development of Threshold Disease. One reason could be that our patients had been followed up for an average of 10 years in both groups in this study. Based on our previous paper, the difference in disease progression between the two groups at the 10-year mark is small and statistically insignificant [18]. This may show that the development of FECD may be affected by other genetic compositions and also external environmental factors [3] that were not analysed in this study.

However, if we look at the percentage of patients who underwent keratoplasty, there was a higher percentage in the L ≥ 40 group compared to the L < 40 group at both follow-up time points. Compared to previous cross-sectional studies, our longitudinal study provides even stronger data to show that patients with CTG repeat expansion are more likely to undergo keratoplasty [21].

Other confounding factors in our study include the duration of follow-up and the small sample size. As this was a prospective cohort study, patients were recruited over a period to gather an adequate number of patients. A post hoc power calculation places this study at 0.0571; however, a sample size required to attain sufficient power at 0.80 totals more than 3000 subjects recruited, which is a near impossible recruitment number given the nature of a longitudinal study. Apart from being able to find a vast number of patients to recruit, it is also challenging to ensure that patients are willing and able to remain in a study for an extended period of time. Having a cohort of patients returning for follow-up over many years (mean time 10 years) can be challenging with respect to compliance. Moreover, it added to the challenge that we excluded patients who subsequently underwent cataract operation during our period of review, as we are aware that ocular surgery can accelerate the clinical disease of FECD [26]. Thus, the duration of follow-up for each patient between the two blood tests varied, which can influence the amount of clinical progression that occurred. However, when we look at the time taken before patients reached Threshold Disease, the patients in the L ≥ 40 group took a shorter time compared to those in the L < 40 group (*p* = 0.127). Another confounding factor is the age of the patients. As we know, age is also a factor that influences the progression of disease in FECD [2]. Thus, the severity of disease may also be influenced by the difference in age. This study was performed only in Asians, in whom the prevalence of CTG repeats is lower as compared to Caucasians [9,11,12,13]; hence, other genetic variants may be implicated, which is an aspect worth looking into for future studies as well. Another consideration for future studies would be to include a patient-reported outcome measure (PROM), such as the V-FUCHS (Visual Function and Corneal Health Status) questionnaire. This would allow us to analyse how patients’ subjective perception of disease corresponds with clinical progression and genetic make-up.

In summary, our study showed that the number of CTG repeats in peripheral leukocytes of FECD patients with fewer than 40 show minimal change over time, and, consequently, patients’ genetic risks also remain largely similar over time. Thus, CTG-repeat expansion targeting therapy is not likely to be required in the future for L < 40, and therapy should focus on other antioxidant defence or gene augmentation of other faulty genes. Our results were not able to show a significant difference in the risks of attaining Threshold Disease between patients in the L < 40 and L ≥ 40 groups; however, more patients underwent keratoplasty in the L ≥ 40 group (*p* = 0.103). By knowing a patient’s genetic risk and how it affects disease progression, clinicians can better counsel patients and better decide on management and follow-up plans [55,56].

## Figures and Tables

**Table 1:** Demographics of 17 patients (27 eyes) analysed for this study.

Demographics		Number of Patients	Percentage
Age	40–49 years	1	5.9%
	50–59 years	10	58.8%
	60–69 years	5	29.4%
	70–79 years	1	5.9%
Sex	Female	10	58.8%
	Male	7	41.2%
Ancestry	Chinese	12	70.6%
	Malay	3	17.6%
	Indian	1	5.9%
	Others	1	5.9%
Number of eyes	Both eyes	10	58.8%
	Left eye	6	35.3%
	Right eye	1	5.9%

**Table 2:** Compiled number of subjects with no expanded allele (SS), 1 expanded allele (SX), and both expanded alleles (XX). TP-PCR + STR gold-standard CTG18.1 genotyping versus computational short-read WGS showed a 100% concordance rate between both methods.

Method	No Expansion (SS)	Expansion in One Allele (SX)	Expansion in Both Alleles (XX)	Total	Concordance
TP-PCR + STR (gold standard)	8	9	0	17	-
Short-read WGS	8	9	0	17	17/17 (100.0%)

**Table 3:** Compiled data from the details of individual subjects recruited for this study. Patients with diagnosed FECD severity of Krachmer Grade 4 and above were recruited for this study after providing informed consent. Eyes with ocular surgery performed prior to genotyping or during the follow-up duration were excluded.

Subject ID	CTG Expansion Status		Time Between Repeat Genotyping (Years)	Eye	Time to Threshold Disease (Years)	Total Follow-Up Time (Years)
	First Genotyping	Repeat Genotyping				
FDC024	SS (12/25)	SS (12/25)	10.7	OS	8.5	16.6
				OD	-	15.0
FUCH 71	SS (12/25)	SS (16/21)	4.8	OS	1.8	8.2
				OD	EXCLUDED	
FUCH 69	SS (12/15)	SS (12/15)	14.2	OS	-	15.8
				OD	EXCLUDED	
FUCH1	SS (18/25)	SS (18/25)	11.9	OS	7.8	13.5
				OD	EXCLUDED	
FUCH 82	SS (12/12)	SS (12/12)	11.0	OS	7.5	12.9
				OD	-	12.9
FUCH 68	SS (12/13)	SS (12/13)	12.9	OS	-	11.9
				OD	-	11.9
FD43	SS (12/17)	SS (12/17)	15.4	OS	15.5	16.8
				OD	4.42	16.8
FUCH 81	SS (18/24)	SS (19/24)	5.1	OS	-	6.6
				OD	-	6.6
FUCH 85	SX (26/>100)	SX (26/>100)	6.5	OS	2.75	9.2
				OD	2.58	9.2
FD07	SX (33/>100)	SX (33/>100)	14.3	OS	6.58	17.7
				OD	EXCLUDED	
FUCH 88	SX (12/90)	SX (12/>100)	4.7	OS	6.25	10.9
				OD	-	10.9
FD062	SX (18/>100)	SX (18/>100)	14.4	OS	2.83	16.6
				OD	-	16.6
FUCH 86	SX (22/>100)	SX (23/>100)	4.8	OS	-	6.9
				OD	-	6.9
FUCH 80	SX (26/>100)	SX (26/>100)	8.8	OS	1	10.7
				OD	4.83	10.7
FUCH17	SX (24/>100)	SX (24/>100)	11.8	OS	EXCLUDED	
				OD	-	14.0
FDC025	SX (26/>100)	SX (26/>100)	15.8	OS	8.75	17.8
				OD	EXCLUDED	
FDC021	SX (26/>100)	SX (26/>100)	13.5	OS	-	19.2
				OD	EXCLUDED	

## Data Availability

The original contributions presented in this study are included in this article/the Supplementary Material. Further inquiries can be directed to the corresponding author.

## Supplementary Materials

The following supporting information can be downloaded at https://www.mdpi.com/article/10.3390/medsci14010031/s1, Supplementary Figure S1: Representative STR/TP-PCR tracings of genotyped samples.

## Abbreviations

FECD (Fuchs’ endothelial corneal dystrophy), ECD (endothelial cell density), CCT (central corneal thickness), STR (short tandem repeat), TP-PCR (triplet primed polymerase chain reaction), WGS (whole-genome sequencing).
