# Supplementary material for: Longitudinal Study of TCF4 CTG Trinucleotide Repeat Length and Disease Severity in Fuchs’ Endothelial Corneal Dystrophy
Source: Med Sci (Basel). 2026 Jan 7;14(1):31. doi: 10.3390/medsci14010031 (PMC12821436; doi:10.3390/medsci14010031)
Supplement: Supplementary file 1 [file medsci-14-00031-s001.zip › medsci-4061367-supplementary.pdf]

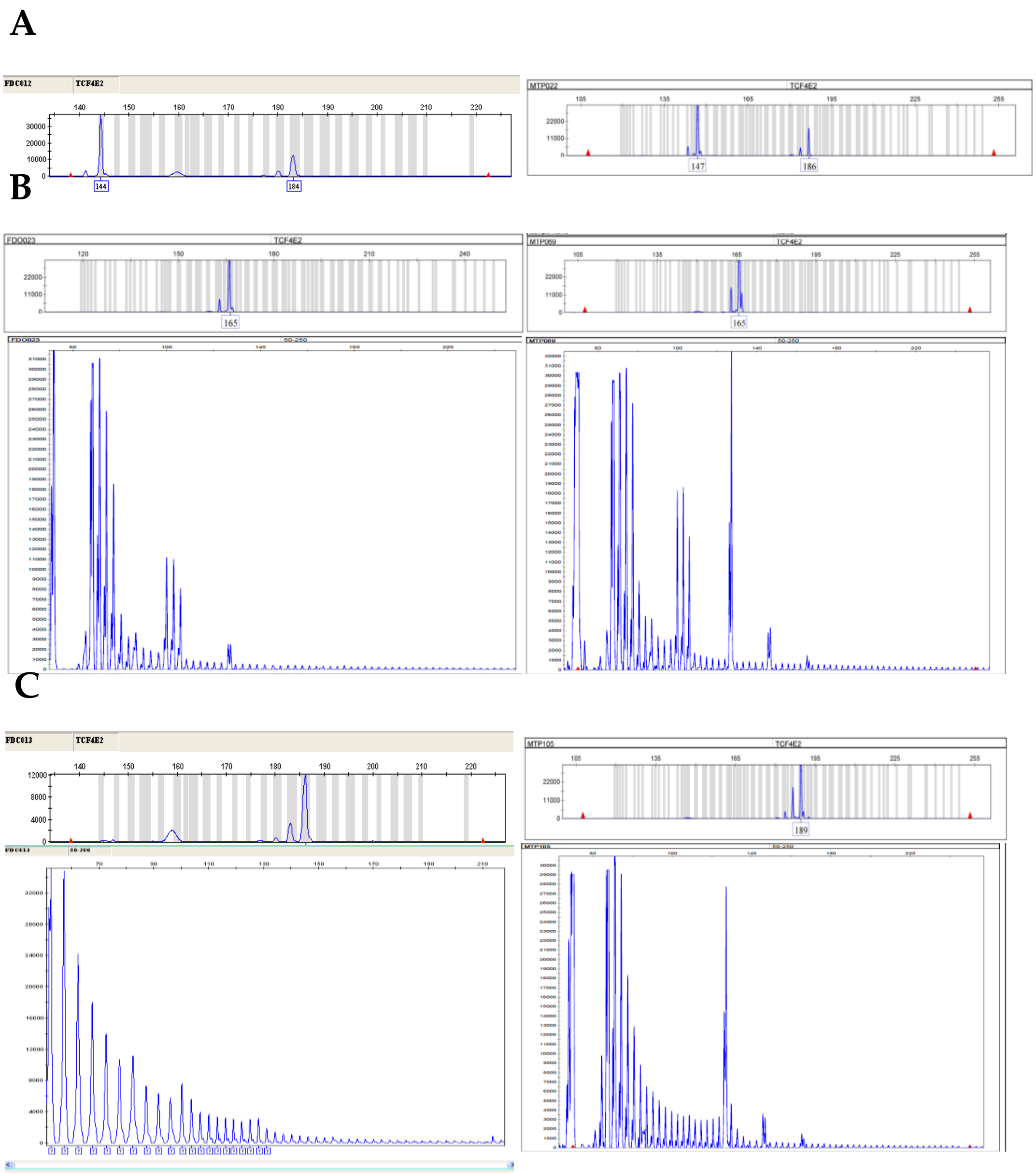

Supplementary Figure S1. Representative STR/TP-PCR tracings of genotyped samples with a non-expanded allele (SS) and one expanded allele (SX). None of the subjects expressed both expanded alleles in this study. Tracings on the left were taken at the first time point, and tracings on the right were taken at the second time point. The number of repeats in each respective allele is (**a**) 12/25, (**b**) 18/>100, and (**c**) 26/>100.
